# Machine learning-based single-sample molecular classifier for cancer grading

**DOI:** 10.3389/fonc.2025.1617898

**Published:** 2025-07-16

**Authors:** Zoia Antysheva, Nikita Kotlov, Mariia V. Guryleva, Ivan Valiev, Viktor Svekolkin, Anna Belozerova, Sheila T. Yong, Dmitry Tabakov, Alexander Bagaev, Vladimir Kushnarev

**Affiliations:** Research and Development, BostonGene Corporation, Waltham, MA, United States

**Keywords:** molecular grade, gene expression, tumor grade, tumor cell differentiation, risk assessment, cancer diagnostics

## Abstract

Tumor subtyping based on morphological grade is used in cancer treatment and management decision-making and to determine a patient’s prognosis. While low- and high-grade tumors are predictive of patient survival for many cancers, tumors of intermediate morphological grades are considered unreliable due to interobserver variability and thus do not have clear prognostic significance. To address this issue, we devised a molecular-based classifier that uses gene expression data from RNA sequencing (RNA-seq) or microarray profiling to predict high- and low-grade risk groups for breast, lung, and renal cancers. For this classifier, we developed a preprocessing procedure that only required expression data from a single sample, without the need for any batch correction or cohort scaling. This classifier, while trained only on RNA sequencing data, achieves highly accurate risk predictions on both RNA-seq and microarray data. First, the molecular grades (mGrades) predicted by this classifier correlated strongly with the pathologist-assigned histological grades and clinical stage. Next, we showed that mGrades were effective in assessing risk levels for G2 samples. Finally, we identified common and unique biological and genetic features in samples of low and high mGrades across breast, lung, and renal cancers. Gene expression patterns as revealed by the classifier can provide useful information for both research and diagnostic purposes.

## Introduction

Tumor grading assesses the differences between tumor and normal tissues, such as tissue de-differentiation and tissue-specific indicators. These differences include combinations of tumor histo- and cytoarchitectonics such as patterns (e.g., solid, tubular, cribriform), number of mitoses, and nuclear and nucleoli pleomorphism, to name a few (1). The use of separate systems for each tumor type allows for a more accurate evaluation of metastatic potential and overall prognosis.

Tumor aggressiveness most commonly ranges from the first to third grade (in breast cancer [BRCA] and lung adenocarcinoma [LUAD]), or, less often, fourth grade (for kidney cancer). Tumors of lower grades are more similar to normal tissues than those of higher grades are. G1 is considered low-grade, G3 and G4 are considered high-grade, and the rest are considered moderate. Tumor grades are considered not only as prognostic and predictive factors, but also as predictors of treatment response. High grades are usually associated with an increased risk to the patient (2–5). The widely accepted Nottingham grading system for BRCA is an independent prognostic biomarker-based grading system that analyzes the degree of tubular formation, mitosis, and nuclear polymorphism (4, 6, 7). It is associated with chemotherapy response (8, 9) and is widely incorporated in clinical guidelines for breast cancer such as those by the American Society of Clinical Oncology (ASCO) (10), National Comprehensive Cancer Network (NCCN) (11), and European Society of Medical Oncology (ESMO) (12).

The newly introduced consensus grading system for LUAD by the International Association for the Study of Lung Cancer (IASLC) showed a significant prognostic impact on survival (3). Tumor pattern has also been reported to affect chemotherapy response (13), whereby the combination of *KRAS* mutation and solid tumor pattern may correlate with chemotherapy resistance (13).

For clear cell renal cell carcinoma (ccRCC), Fuhrman grading (14) implemented in clinical models to predict recurrence had been the gold standard for tumor grading. After a consensus meeting in 2012, the International Society of Urological Pathology (ISUP) replaced the Fuhrman grading system with a new grading system, WHO/ISUP (5). High-grade ccRCC tumors based on the WHO/ISUP grading system (15) showed a correlation not only with poor prognosis, but also with non-responders to sunitinib (16).

Despite the prognostic and predictive importance of tumor grading, its application is challenging. First, reproducibility is the main concern. Since this analysis is performed by a pathologist, there is often substantial variation in the analysis outcome (5, 17, 18). Thus, despite the use of various criteria to maximize assessment objectivity, the definition of tumor grade remains subjective and highly dependent on user expertise (19). Moreover, there is a lack of statistically significant differences in the survival rate of patients with moderate-grade tumors when compared to patients with low- and high-grade tumors (20).

Compounding this problem is the fact that pathologists often assign samples as G2. As such, it stands to reason that the G2 grade is uninformative with regard to clinical decision-making and its prognostic value is uncertain (19, 21, 22). Machine-learning approaches have been shown to perform better at risk prediction in cancer sample analysis than many other existing approaches (23). Unfortunately, the development and use of machine-learning approaches for grade assessment do not completely resolve the issue of reproducibility (4, 18). This is because although deep learning and machine learning can render an algorithm or model capable of predicting tumor grades, these approaches still require validation, often by pathologists (24). Pathologists performing the validation steps are often also responsible for annotating the training dataset for the model; hence, they are likely to subconsciously incorporate their own biases into the process (25, 26). Therefore, it may be technically impossible to eliminate all biases.

One of the most successful attempts to mitigate this problem was the creation of the Genomic Grade Index (GGI) (21). In this study, GGI was defined as the difference between the sum of the expression of genes with increased expression in G3 and the sum of the same parameters in G1. The ability to divide patients with index values (above or below zero) into groups of high and low malignancy was shown. The resulting separation and index value were associated with survival, with a high index value associated more strongly with a higher risk of recurrence than a low gene expression grade index value, based on pathological analysis (hazard ratio = 3.61, 95% confidence interval = 2.25 to 5.78; p<.001, log-rank test). Notably, the authors used GGI to divide samples of intermediate malignancy (G2) into two groups corresponding to low- and high-grade malignancies. Although powerful, GGI still requires a cohort of samples belonging to a single batch and enriched in both low- and high-grade samples for initial scaling before it can be deployed to analyze other clinical/test samples belonging to this specific batch.

Many existing predictors can only use one type of data (RNA sequencing [RNA-seq] or microarray data) and can only perform cohort analysis (27). This is a serious limitation in the clinical setting because in order to make educated decisions concerning treatment options and to predict responses, we need to be able to analyze single patient samples without the need for a cohort. Currently, most of the developed predictors are created for individual nosological cancers (28). Despite the active development of predictors in recent years, achieving clinically significant predictive accuracy and applicability of a predictor in clinical practice remains a challenge. To address this challenge, we developed a molecular-based classifier that can use gene expression data from either RNA-seq or microarray profiling to accurately predict the tumor grades for BRCA, LUAD, and ccRCC. This approach aligns with recent efforts to enhance molecular stratification across diverse cancer types, including pancreatic cancer, where molecular insights into tumor microenvironment and signaling pathways are actively shaping therapeutic strategies (29).

## Results

### Development and training of the tumor grade predictor

**Figure 1:**
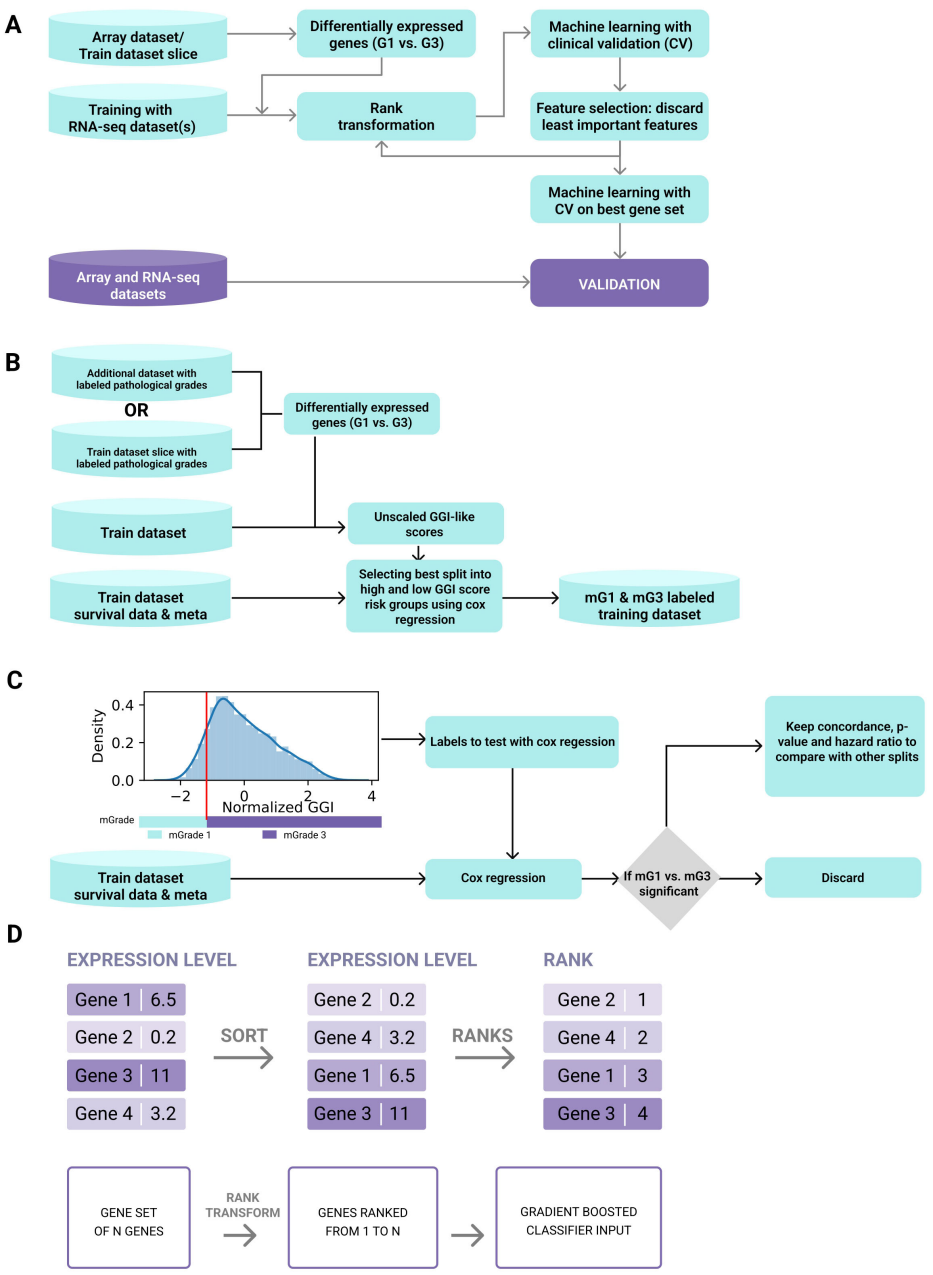
Development pipeline of the tumor grade predictor. **(A)** The tumor grade predictor was developed by training a machine-learning algorithm to classify tumors into categories of low (mG1) and high (mG3/mG4) molecular grades (mGrades) for each cancer type using rank transformation of gene expression data from multiple datasets. **(B)** Scheme for selecting the best split into risk groups of high and low GGI scores using Cox regression. **(C)** Scheme for optimal threshold definition. **(D)** Rank transformation was used to conserve gene relationships (i.e., GeneA > GeneB) and transform geneset values into fixed ranges for a single sample, independent of dataset composition.

The tumor grade classifier was developed by training a machine-learning algorithm to classify tumors into low (mG1) and high (mG3/mG4) molecular grades (mGrades) for each cancer type using rank transformation of gene expression data from multiple datasets (**Figure 1A**). We developed this tumor grade predictor with three key objectives. First, we aimed to accurately differentiate between high- and low-grade tumors. Second, we aimed to efficiently stratify samples with intermediate tumor grades, such as G2, into either high- or low-risk categories. Lastly, we wanted to perform tumor grade prediction for single patient samples, without the need for large cohorts or complex procedures.

To achieve these objectives, we used gene expression data for BRCA, LUAD, and ccRCC from publicly available datasets (see Materials and Methods and **Supplementary Table 1**) and developed a classifier for each cancer type. Each classifier was trained only on the RNA-seq data, and then validated using both microarray and RNA-seq data. To label a dataset for classifier training (**Figure 1B**), we performed differential expression analysis between high- and low-grade samples either on a subset of this dataset or on an additional microarray dataset that would subsequently be excluded from the validation step. Genesets differentially expressed between G3 (or G4) and G1 tumors were used to create a gene expression grade index (GGI) for all samples in the training dataset, as described by Sotiriou et al. (21) (**Supplementary Figure S1A**). However, unlike the original study, our GGI was not scaled using pathologist-labeled grades. Instead, we employed a vector of unscaled GGI values in survival analysis using Cox regression. In this analysis, the samples were stratified into high- and low-GGI groups based on a predetermined threshold. This threshold was refined by testing potential cutoffs at 1% intervals of the GGI variance and selecting the one that provided the best p-value, hazard ratio for grade groups, and overall concordance (**Figure 1C**). This approach enabled threshold optimization without relying on external grading, ensuring that the model could independently predict risks based on molecular data alone.

The training dataset for each cancer type was split into high- and low-risk groups based on GGI values and publicly accessible survival data for each cancer type. We defined these risk groups as molecular grades (mGrades) and labeled the low-risk group as mG1 and high-risk groups as mG3 or mG4 depending on the cancer type. As expected, most of the high-grade samples clustered in the high-risk group, whereas most of the low-grade samples clustered in the low-risk group. Samples with G2 or other intermediate grades and unlabeled samples were split between the two risk groups (**Supplementary Figures S1A, B**). The sample labels were then used to train the classifiers.

To train the respective models for each cancer type, we used only the differentially expressed genesets that were used for GGI calculations. Rank transformation, as described in the Materials and Methods section, was used to conserve gene relationships (i.e., GeneA > GeneB) and transform gene values into fixed ranges for a single sample, independent of dataset composition (**Figure 1D**). This approach stabilizes the tree classifiers that use rules such as GeneA > ValueB, allowing single-sample classification to be independent of batch and dataset composition. Therefore, unlike conventional batch correction methods (such as ComBat), rank transformation does not provide true batch correction because it does not deliver continuous values for all genes or large genesets (30). Nonetheless, for single-sample corrections performed in this study, rank transformation was sufficient to enable batch-independent sample classification.

Next, we performed machine-learning feature selection to choose the best geneset for mGrade classification. The SHAP values of feature importance (31) were used to determine and discard the least important genes (see Materials and Methods). The rank transformation step was repeated for the expression data. The model was then trained on the refined feature set. This step was repeated until 20 features remained, followed by the selection of the geneset with the best negative log loss (**Supplementary Figure S2A**). This approach revealed the strongest positive contributors across all three cancer-specific models to be *BIRC5, TPX2, CTHRC1, SLC7A5*, and *MMP7*. The convergence of cell-cycle regulator genes (*BIRC5, TPX2*) with matrix-remodeling and metabolic genes (*CTHRC1, SLC7A5, MMP7*) underscores the twin biological hallmarks—unchecked proliferation and micro-environmental restructuring—that distinguish high (mG3/mG4) from low (mG1) molecular grades.

Upon selecting the best geneset (**Supplementary Figure S2B**), we repeated the rank transformation step with this geneset and then trained a final model with hyperparameters optimized by cross-validation. Finally, we validated our models using cancer type-specific microarray and RNA-seq datasets (**Supplementary Table 1**).

### Model validation and testing

The validation datasets (**Supplementary Table 1**) were used to assess model performance. Two criteria were used to evaluate model quality. The first was the capability of the classifier to discern low and high histological grades based on gene expression data, thus confirming the association between molecular and histological grades. Second was the capability of the mGrades to predict survival and to split samples into low- and high-risk categories, especially for samples with intermediate histological grades.

**Figure 2:**
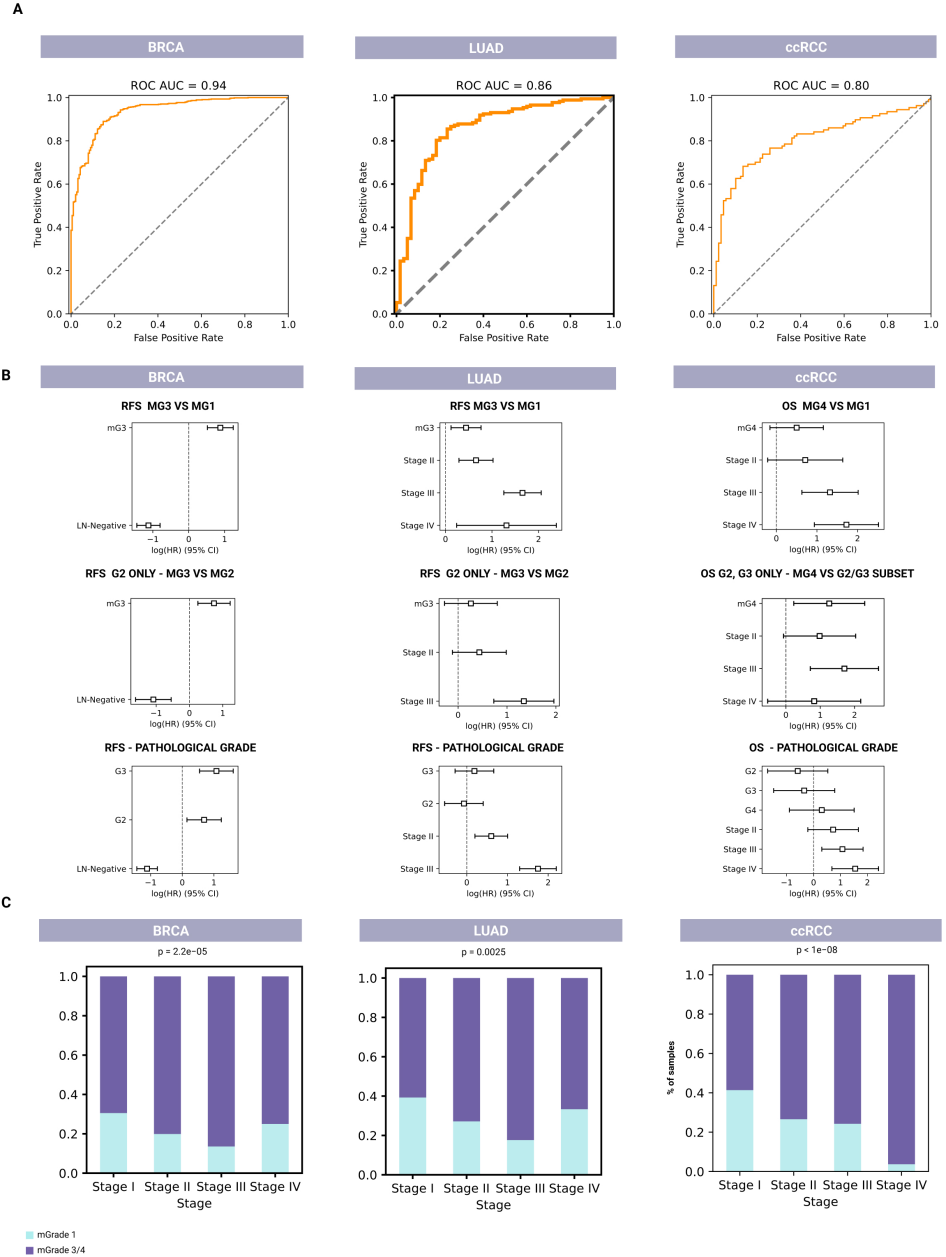
Molecular-based classifiers for predicting tumor grade and survival for BRCA (left, n=1,927), LUAD (middle, n=596), and ccRCC (right, n=718). **(A)** AUC ROCs for pathological classification of the mGrade predictor to demonstrate the accuracy of pathological classification of G1 and G3/G4 samples into mG1 and mG3/mG4 groups using RNA-seq and microarray data. **(B)** Cox regression analysis plots for comparing the prognostic values of mG1 and mG3 groups (top row) to histological grades (bottom row). Middle row: Cox regression analysis plots showing the prognostic value of mGrade samples after the splitting of G2 pathological samples by the classifier. **(C)** Bar graphs showing the association of mG1 and mG3/mG4 groups with clinical stage across all three cancer types.

As shown in the area under the receiver operating characteristic curves (AUC ROCs) in **Figure 2A**, our mGrade predictor classified BRCA, LUAD, and ccRCC samples into mG1 and mG3/mG4 groups with high accuracy based on RNA-seq and microarray gene expression data. The AUC exceeded 0.8 for all cancer types. In particular, BRCA, which had the highest number of samples analyzed, demonstrated an AUC ROC value of 0.936. The regression concordance values obtained from Cox regression survival analysis showed that our predictor performed at least as well as histological grades in predicting survival (**Figure 2B**; **Supplementary Tables 2–10**). Additionally, our predictor could split samples with histological grade G2 and other intermediate grades into high- and low-risk groups. We also found that mG3 had a higher hazard ratio than histological G3 for LUAD and ccRCC (**Figure 2B**), thus allowing our mGrade predictor to classify high-risk samples and capture high-risk patients more effectively. In other words, this predictor can identify high-risk patients more precisely than histological grading can. This is an important feature of our classifier for addressing the prognostic uncertainty of the G2 grade, as it has been demonstrated previously that samples with intermediate grades could in fact be divided into high- and low-risk categories (21). In this regard, the mGrade predictor offers an objective and unbiased means to classify these samples into the mG1 or mG3/mG4 groups.

Moreover, Cox models that used mGrades achieved concordance indices equal to or higher than those based on traditional histological grades in all three cancer types (BRCA, LUAD, and ccRCC). The integrated Brier score for the mGrade model differed from the histology model by no more than 0.002. Importantly, when G2 tumors were reclassified into mG1 or mG3, the resulting two-class model produced a lower (better) Brier score than the original three-grade histology model (**Figure 2B**; **Supplementary Tables 2–10**). Importantly, our approach described herein uses a GGI-like index based on differential gene expression and survival data that often reassigns certain histologically labeled G1 and G3 samples to different risk groups if their molecular profiles diverge from the conventional morphological classification. Because these new molecular labels do not fully align with the original pathologist-assigned grades, complete agreement between our GGI-based labeling and traditional histological labels is inherently impossible. It is also noteworthy that concordance between pathologists is not absolute even when G1 and G3 grades are concerned (32). Since pathological label prediction is not absolute and is not a training objective, it is not unusual for samples to be assigned a different grade from the initial pathological labeling.

Moreover, our analysis showed a significant association between cancer stage and mGrades (mG1 and mG3/mG4) in BRCA, LUAD, and ccRCC (p < 0.005). In BRCA and LUAD, an increased proportion of mG1 samples was observed in Stage IV, marking a shift from the expected trend of the mG1 proportion decreasing and the mG3/mG4 proportion increasing with advancing stage. In ccRCC, this trend remained consistent across all stages, with a decrease in the proportion of mG1 samples and an increase in the proportion of mG3/mG4 samples as the disease stage progressed (**Figure 2C**). The different mGrade trends in Stage IV of BRCA and LUAD can be explained by the low number of Stage IV samples of these two cancers in the validation set (20 and 6, respectively).

Models that analyze designated sample batches have also been reported (33). Unfortunately, due to the presence of batch effects, these models cannot be used to analyze single samples or samples from different batches. Therefore, their application is mostly limited to research settings and is not easily translatable to clinical practice. While there have been attempts to develop single-sample predictor models and approaches, they typically focus on only one cancer type (27, 28). In contrast, our classifier was developed as a generalized method to analyze single samples of multiple cancer types, which allows model customization for specific cancer types.

Using validation datasets and the datasets outlined in **Supplementary Table 1**, we aimed to elucidate the distinct features and biological processes that characterize tumor grades across various cancer types. This approach allowed us to uncover the biological underpinnings of the mGrades, thereby enhancing the utility of our mGrade predictor.

### Breast cancer (BRCA)

To examine the mGrades for BRCA more closely, we split the best geneset selected for the classifier into groups of genes upregulated in G1 and G3, according to the outcome of our differential gene expression analysis described in the Materials and Methods section. Each group was examined for enriched pathways (**Supplementary Figure S4B** and **Supplementary Table 11**).

First, we found that the mG3 gene group was highly enriched in pathways associated with proliferation and cell cycle regulation. This finding is consistent with the definition of high tumor grade in breast cancer, which features a high mitotic count and pleomorphic nuclei (6). The mG1 group showed enrichment in the angiotensin II receptor pathway, indicative of angiogenesis and microenvironmental changes.

**Figure 3:**
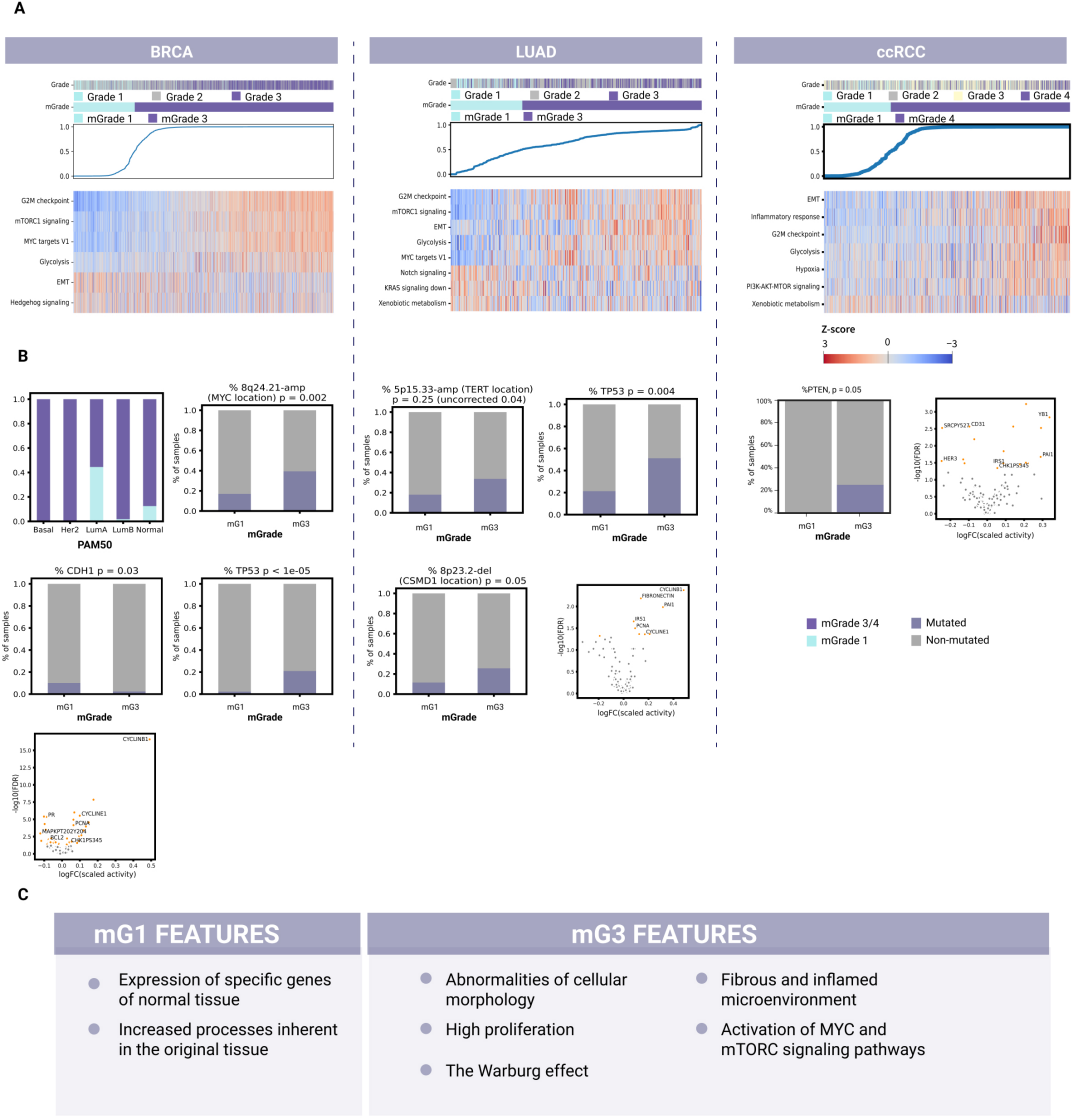
Identification of common and unique biological features for each mGrade across BRCA, LUAD, and ccRCC samples. **(A)** Heatmaps showing the split of histological grades into mGrades (mG1 and mG3/mG4), characterized by different pathway activities in BRCA, LUAD, and ccRCC. Blue line: model-predicted mGrade probability for each sample (in ascending order). The color key applies to the entire panel **(A)**. **(B)** Differential mutations, CNA regions, protein expression, and molecular subtypes (BRCA) for BRCA (left), LUAD (middle), and ccRCC (right). **(C)** A list of common biological features of mG1 and mG3 samples identified across BRCA, LUAD, and ccRCC samples after evaluating common pathway activities, geneset enrichment, mutations, copy number alterations, and differentially expressed proteins.

Interestingly, the best geneset selected also included six genes associated with cellular morphology, including those involved in cell cilium maintenance: *WDR19*, *KIF13B*, and *IFT88*. These cell cilium maintenance genes were downregulated in mG3. Cell cilium loss has been previously reported as an early event in breast cancer progression and is one of several cellular abnormalities defined by a high tumor grade (34). Moreover, cell cilia play a role in regulating Sonic Hedgehog (SHH) signaling, since their loss reportedly leads to reduced SHH signaling in tumorigenesis (35). In agreement with these published findings, our ssGSEA (single-sample gene set enrichment analysis) scores also revealed a statistically significant downregulation of SHH signaling in mG3 samples that showed a loss of cell cilia (**Figure 3A**; **Supplementary Figure S4A**, and **Supplementary Table 12**).

Moreover, our ssGSEA scores on validation data further confirmed the connection between mG3 and high proliferation, since Hallmark_G2M_Checkpoint and other pathways associated with the cell cycle were significantly upregulated. Another feature of interest is the activation of glycolysis in mG3 samples, indicative of metabolic changes that switch cells in high-grade breast cancer tumors from aerobic to anaerobic respiration. mG1 samples had upregulated pathways associated with epithelial-mesenchymal transition (EMT) and apical junctions, indicative of abnormalities in cellular morphology regulation, cell contact with the extracellular matrix (ECM), and cell motility (**Figure 3A**; **Supplementary Figure S4A**, and **Supplementary Table 12**).

Next, we analyzed the TCGA-BRCA cohort to assess the transcriptomic, proteomic, and genomic data. Here, we found that the PAM50 subtype groups Basal and Her2 consisted almost exclusively of mG3 samples (**Figure 3B**). These subtypes are known for their high proliferation rate and aggressiveness (36, 37). As these subtypes are highly different from the Luminal and Normal-Like subtypes, their presence will confound the analysis of mG3 vs mG1, possibly leading to erroneous or loss of definitions of common features for mG1 and mG3. Therefore, we excluded them from further analysis of mGrade properties for TCGA-BRCA to avoid additional confounding factors so that we could capture the differences between mG1 and mG3 more clearly.

Our analysis of the genomic data of TCGA-BRCA revealed that the mutation rate of *CDH1* was higher in the mG1 samples. *CDH1* (encoding E-cadherin) is frequently mutated or lost in cancers, including breast cancer (24, 38, 39). Loss of E-cadherin, one of the most well-known cell contact components, is associated with EMT in tumor cells and metastasis (40). Our data also suggest this to be the case for mG1 samples, since the Hallmark_EMT signature was significantly upregulated in mG1 samples (**Figure 3A**). There was also an enrichment of *PIK3CA* and *MAP3K1* mutations in mG1 samples, indicating the specificity of this mechanism in tumor development (**Figure 3B**; **Supplementary Figure S3A**, and **Supplementary Table 13**). In a recent publication, we reported that mG1 tumors are often associated with Luminal A (LumA) breast cancers, whereas mG3 tumors are frequently observed among triple-negative breast cancers (TNBC) and Luminal B subtypes (41). LumA tumors often show activation of the PI3K/AKT/mTOR and MAPK/ERK signaling pathways, which are associated with *PIK3CA* and *MAP3K1* mutations, respectively (42, 43). These observations may explain the enrichment of these mutations in mG1 tumors, which primarily belong to the LumA subtype.

We also found that *TP53* mutations were more common in mG3 tumors. Such mutations may contribute to the high proliferation rate of these tumors, as *TP53* encodes p53, a well-known tumor suppressor that stops the cell cycle at the G1/S phase and initiates apoptosis when DNA damage becomes too extensive (39, 44).

Analysis of genome alterations revealed a large number of segments that were altered differently among the different mGrades. The most significantly altered segments in breast cancer included 11q13.3-Amp where mitogene *CCND1* is located, and 8q24.21-Amp where oncogene *MYC* is located (**Figure 3B**; **Supplementary Figure S3B**, and **Supplementary Table 15**). These genes promote cell proliferation and survival and are among the most commonly amplified genes in breast tumors (39, 45).

Furthermore, the differential protein expression observed also supports the description of mG3 as a highly proliferative class. Among the proteins with the highest log-FC, cyclin B1 was upregulated in mG3 tumors (**Supplementary Table 16**). Cyclin B1 regulates mitosis as a complex with Cdk1 (46) and is activated by c-MYC and inhibited by p53, which is consistent with *c-MYC* amplification and *TP53* mutations observed in mG3 tumors (**Figure 3B**).

Based on these findings, we postulate that mG3 breast tumors are highly proliferative, and that their proliferation is stimulated through mTOR and c-MYC signaling. These cells often suffer from p53 loss, which further dysregulates the cell cycle. Other common properties of mG3 breast tumors include the loss of normal cell morphology, increased aggressiveness, and a switch to anaerobic respiration. Conversely, mG1 tumors retain normal-like morphology and do not lose cell cilia or SHH pathway activity. mG1 cancer cells also seem more prone to EMT, possibly because of mutations in *CDH1*.

### Lung adenocarcinoma (LUAD)

The best geneset selected by the classifier was also examined for pathway enrichment in LUAD (**Supplementary Figure S4B** and **Supplementary Table 17**). The first group of enriched pathways in mG3 tumors consisted of those connected to integrins, which are involved in regulating cell morphology, contacts, and motility. The second group consisted of pathways related to regulation of microenvironment and fibrin clotting. Pathways associated with proliferation were also enriched. Moreover, the ssGSEA scores revealed upregulation of proliferation and glycolysis pathways in mG3 tumors (**Figure 3A**; **Supplementary Figure 4A**, **Supplementary Table 18**). The upregulation of Cyclin B1, Cyclin E1, and PCNA proteins was indicative of increased proliferation in mG3 tumors (**Figure 3B**). In mG1 tumors, the p53 and Notch signaling pathways were upregulated, while KRas signaling was downregulated. Notch signaling is active in normal lungs, where it influences cellular fate during differentiation (47). This is consistent with mG1 being the less dedifferentiated class. KRas signaling is one of the driver pathways of LUAD. This pathway can also activate the PI3K-AKT-mTOR signaling axis; indeed, the observed ssGSEA scores indicated an upregulation of mTORC signaling in mG3 tumors.

To discover other features of mGrades for LUAD, we analyzed 111 designated holdout samples of TCGA-LUAD and 38 PCAWG samples with mutation data. One of the genomic features that differed most prominently between mG3 and mG1 was *TP53* mutation rate (**Figure 3B**; **Supplementary Figure S3A**, and **Supplementary Table 19**). *TP53* was more commonly mutated in mG3 samples, demonstrating the genomic basis behind the upregulation of proliferation and downregulation of the p53 pathway in mG3 tumors. Meanwhile, analysis of copy number alterations (CNAs) revealed a deletion in the 8p23.2 region (the *CSMD1* locus) in mG3 (**Figure 3B**; **Supplementary Figure S3B**, and **Supplementary Table 20**). *CSMD1* is a tumor suppressor gene implicated in both EMT regulation and cell development, and influences proliferation and apoptosis (48). The increase in EMT and pro-metastatic processes is in line with the EMT signature upregulation in mG3 tumors. Interestingly, 8p23.2 was also more frequently deleted in BRCA mG3 tumors although the EMT signature was upregulated in mG1 (**Supplementary Tables 12, 14**), suggesting that *CDH1* inactivation and *CSMD1* deletion might be two independent mechanisms for tumor metastasis. Another trend, although not significant after FDR correction, was the amplification of the 5p15.33 region where *TERT* (encoding telomerase reverse transcriptase) is located. Telomerase reverse transcriptase facilitates cancer growth and cell survival and is overexpressed in many cancers, including LUAD (49). Our observations corroborate previously published findings implicating disruption of the *RB1*/*CDKN2A*/*TP53* axis in the G1/S phase of the cell cycle and apoptosis checkpoints as an early tumor-initiating event in *EGFR*-mutant LUADs (50).

mG3 tumors also demonstrated an inflamed microenvironment prone to EMT, as evidenced by enrichment of the best classifier genes featuring ECM organization pathways and collagen rearrangement, upregulation of the EMT signature, and the aforementioned *CSMD1* deletion. In line with these findings, protein analysis also revealed upregulation of PAI1 and fibronectin in mG3 (**Figure 3B**; **Supplementary Table 22**). PAI1, encoded by *SERPINE1*, is an inhibitor of fibrinolysis. It may play a role in forming a fibrotic and inflamed microenvironment as the tumor progresses (51).

Thus far, our analysis has depicted mG3 LUAD tumors as highly proliferative; their proliferation may be activated through Ras signaling and the PI3K-AKT-mTOR axes. Mutations in *TP53* were also consistent with the proliferative qualities of mG3 tumors. mG3 tumors are prone to microenvironment and cell contact remodeling, as indicated by the enrichment of pathways involved in integrin and fibrin regulation. They may be predisposed to EMT, as supported by *CSMD1* deletion and active microenvironment remodeling. Meanwhile, mG1 tumors are associated with the activation of Notch signaling and pathways related to ROS and xenobiotic metabolism, all of which play important roles in normal lung tissues.

### Clear cell renal cell carcinoma (ccRCC)

Of the pathways enriched in the geneset for mG3 clear cell renal tumors, those describing immune system activity are among the most abundant (**Supplementary Figure S4B** and **Supplementary Table 23**) (52). This suggests the presence of inflammatory processes in these tumors. Also abundant are pathways associated with fibrosis and ECM remodeling in the microenvironment, including processes related to coagulation and the complement system in the immune response. These findings suggest the presence of a fibrotic and inflamed microenvironment in tumors with high mGrades. Notably, inflammation in ccRCC is widely associated with worse survival and a lack of therapeutic response (53).

Both pathway enrichment and ssGSEA scores for mG3 tumors demonstrated the activation of Hallmark_EMT in these tumors (**Figure 3A**; **Supplementary Figure S4A**, and **Supplementary Table 24**). EMT is associated with metastasis, while fibrosis is associated with both EMT and inflammation. Thus, we postulate a greater risk of metastasis in mG4 tumors than in mG1 tumors.

In agreement with previous findings, the Warburg effect in which tumor cells switch from cellular respiration to glycolysis as their main energy source was also apparent in our analysis of ccRCC cells (54). While mG1 cells showed active gluconeogenesis, mG4 cells showed hypoxia and glycolysis (**Figure 3A**; **Supplementary Figure S4A**, and **Supplementary Table 24**). Hypoxia stimulates fibrotization in clear cell renal tumors (55), which further supports our classification of mG4 tumors as fibrotic. Additionally, pathways associated with proliferation were enriched in mG4 renal tumors. The increase in proliferation and the presence of the Warburg effect were confirmed by the ssGSEA scores of the validation data (**Figure 3A**; **Supplementary Figure S4A**, and **Supplementary Table 24**). Similarly, the ssGSEA scores confirmed the activity of inflammatory processes in these tumors.

The genes selected for mG1 tumors were enriched in pathways related to transmembrane transport and normal kidney function. This observation was expected given the resemblance of low-grade tumors to normal tissues. Meanwhile, mG4 tumors showed elevated levels of phosphorylated Chk1 according to RPPA array analysis (**Figure 3B**; **Supplementary Table 28**). These findings concur with the activation of proliferation pathways in these tumors. We observed similar findings in high-grade breast tumors.

Interestingly, protein levels of the *SERPINE1* gene were also increased in mG3 ccRCC tumors (**Supplementary Table 28**). This observation supports our earlier findings on pathway activity and enrichment, characterizing mG4 tumors as having an inflamed and fibrotic microenvironment. Notably, an increase in *SERPINE1* expression was also observed in high-grade LUAD tumors (**Figure 3A**; **Supplementary Table 22**). Meanwhile, CD31 levels were increased in mG1 ccRCC tumors, indicating stronger adhesion to the epithelium and a different structure of cellular junctions compared to mG4 tumors (**Supplementary Table 28**). Our analysis showed that mG3 clear cell renal tumors were inflamed and fibrotic. Pathway enrichment analysis revealed that these tumors were prone to EMT activation and were thus more likely to metastasize. Moreover, the Warburg effect and hypoxia observed in these tumors may promote fibrosis. Additionally, mG4 ccRCC tumors showed high proliferation, which was likely promoted by the PI3K-AKT-mTOR axis (**Figure 3A**). In contrast, mG1 tumors retained more properties of normal tissue at the molecular level, as evidenced by the high expression of genes typically expressed in normal kidneys.

## Discussion

The current gold standard for tumor grading is pathological assessment. The critical nature of tumor grading in the management of breast, lung, and kidney cancers is well-documented, as tumor grading is a fundamental component in diagnostic processes and subsequent treatment decision-making. This is supported by extensive literature, including studies on the future direction of grading invasive breast carcinoma (4), the effectiveness of first-line immune checkpoint inhibitors in advanced renal cell carcinoma (56), and grading standards for renal cell carcinoma (57) and lung cancer (3, 58).

Unfortunately, this approach depends strongly on the observations and expertise of pathologists, rendering it highly subjective. As a result, assessment outcomes can vary greatly, leading to suboptimal treatment decisions, inaccurate prognoses, and unreliable prediction of treatment outcomes. Another disadvantage of pathological assessment is the presence of intermediate tumor grades, which do not have clear clinical significance and are difficult to interpret.

There are currently several well-known prognostic tests for BRCA classification, including Oncotype DX, EndoPredict, and GGI (59). Oncotype DX uses an intermediate risk-level to assess BRCA samples. The final score (from 0 to 100) is calculated based on the expression of 21 genes, and the score is used to predict disease recurrence and project benefits from chemotherapy. An intermediate level is interpreted as a medium risk of recurrence. Regarding the benefits of chemotherapy, the scores are divided into two levels. For scores between 16 and 20, Oncotype DX classifies the benefits of chemotherapy as unlikely to outweigh the risks of side effects. Conversely, for scores between 21 and 25, the benefits are likely to outweigh the risks. Regarding risk assessment, intermediate tumor grades are difficult to interpret because of their tenuous association with treatment outcomes and survival. Similarly, tumors of intermediate grades are also challenging to classify based on their biological processes because the differences can vary widely, with overlapping features with high- and low-grade tumors. This phenomenon compounds the analysis of biological processes that may contribute to cancer development and progression, rendering it difficult to stratify patient samples into high- and low-risk groups. EndoPredict analyzes gene expression patterns in breast tumors to stratify the risk of distant metastasis into two levels (low and high) (60). This test tends to place patients in the high-risk group more often than other predictors, possibly due to the limited number of genes analyzed (27). Finally, GGI only accounts for the sum of expression trends for all analyzed genes rather than the trend for individual genes, thereby disregarding the unique contribution of individual genes to the observed pathology. This can lead to a lack of interpretability and may interfere with our understanding of the contribution of each gene to the development and progression of aggressive tumors.

In this study, we developed a pipeline for training molecular-based classifiers to determine tumor grades of samples with different pathologies. These classifiers use a machine-learning approach to predict tumor grades from either RNA-seq or microarray profiling data of cancerous tumors. By applying our pipeline to BRCA, LUAD, and ccRCC, we demonstrated the capability of our algorithm to determine tumor grades regardless of data type (from RNA-seq or various microarrays from different vendors). Additionally, unlike other models (21, 27), predictions by our algorithm were performed independently of sample storage types, preparation protocols, and sample processing sites. This flexibility resulted in an increased data volume for the development and further application of our pipeline.

Another advantage of our tumor grade predictor over most existing predictors that only analyze data from patient cohorts is the utilization of rank transformation that enables the analysis of single samples, rendering our predictor potentially useful for grading tumors in clinical settings. Importantly, the mGrade predictor demonstrated greater accuracy than other single-nosology and single-sample predictors (27, 28) and could split G2 samples into high- and low-risk groups, improving patient stratification. Therefore, the use of our tumor grade predictor will enable physicians to obtain accurate information concerning tumor grade and risk prediction, consequently aiding treatment decision-making and improving patient care. Similar to other models (61), this tumor grade predictor divides intermediate grades into two levels, each associated with prognosis, treatment outcomes, and cancer progression. It showed comparable hazard ratios to histological grades in predicting survival for BRCA, LUAD, and ccRCC (**Figure 2B**) and effectively stratified G2 samples into risk groups. Simplifying the classification of clinical samples into two distinct mGrades (low and high) facilitates a clearer prognostic and predictive understanding, circumventing the ambiguities and challenges posed by intermediate grades. This binary classification not only aids in decision-making, but also allows for a focused examination of biological features without the discrepancies typically introduced by pathological interpretations or the uncertain biological significance of intermediate grades (62, 63). Additionally, the adoption of AI technologies enhances reproducibility and reduces uncertainties in grading assessment, further refining diagnostic accuracy (5, 22). Taken together, these enhancements in tumor grading may facilitate the development of new therapeutic strategies, novel drugs, and improved treatment standards.

Exploration of the molecular characteristics defining tumor grades has also revealed the biological complexities of tumors, as reflected in their biomarker profiles (**Figure 3C**). For instance, the prediction of Oncotype DX scores (64) of breast cancer samples, stratification of RCC patients to appropriate drug therapies (56), and surgical interventions for LUAD (65) are directly influenced by tumor grade. Such discoveries also extend our understanding of the genetic and molecular drivers of tumor progression and malignancy, such as the predominance of the Warburg effect in high-grade renal cancers (66) or the dissemination patterns of micropapillary components in high-grade LUAD resulting from the loss of anchorage molecules (67, 68).

To this end, we analyzed BRCA, LUAD, and ccRCC multi-omic data to demonstrate the utility of mGrades in research, revealing features not captured by pathological grading, such as EMT, SHH signaling (BRCA), proliferation and Notch signaling (LUAD), and the Warburg Effect (ccRCC). High-grade tumors were associated with increased proliferation, glycolysis, and activation of the PI3K-AKT-mTOR axis across all tumor types (**Figure 3A**) (69). The Warburg effect was prevalent in mG3/mG4 tumors, marked by upregulated glycolysis and hypoxia signatures (70). Additionally, fibrotic and immunosuppressive microenvironments were present, despite the abundance of immune cells (71). These findings underscore the potential of our tumor grade predictor to enhance grading precision and reveal key tumor characteristics for therapeutic targeting.

Our current models have some limitations. A key limitation of the present work is that all validation is retrospective; prospective clinical studies are needed to confirm real-world utility. Their performance depends on the dataset size, with larger sample cohorts yielding better predictions. To address this, one can modify the training procedure to include more datasets because our models do not require all samples to come from the same cohort. As new data become available, models can be retrained for other cancer types. A sufficient number of labeled samples (G1 and G3/G4) is required to generate accurate feature sets and gene lists. Poorly defined grades or high pathologist discordance can reduce model effectiveness, which is a challenge shared by other grading models. However, our predictor is more flexible and requires only the highest and lowest grades for training.

Despite these limitations, the mGrades depicted by our grade predictor reflect a summation of biological processes, including proliferation, nuclear organization, and invasiveness into the tumor microenvironment. In our pursuit of advanced tumor grade prediction, we have seamlessly integrated histopathological data with molecular characteristics, harnessing the rich detail offered by traditional pathology and the precision of molecular biomarkers. The implications of these findings are profound because they pave the way for the development of novel therapeutic strategies targeting the most suitable patient demographics based on the identified characteristics of low and high mGrades.

## Conclusion

The developed model shows great potential for use in clinical practice, both for the cancer types described herein and for additional cancer types following model adaptation. The use of this model will enable physicians to move away from the uncertainty of intermediate grades and achieve operational predictions of disease outcomes. The prognostic ability of the mGrades predicted by our classifiers, along with their association with clinical stage, demonstrates their capability to assess the risk levels for G2 samples, and suggests the potential for molecular-based tumor grade classifiers to be used in clinical practice and clinical trials. The mGrade classifier can be integrated into current molecular workflows as a single-sample adjunct test performed on routine RNA-seq data. Once analytically validated in a CLIA/CAP setting, it could be reported alongside conventional biomarkers to eliminate the ambiguity of intermediate grades and support treatment selection. Prospective interventional trials will be required to establish clinical utility and cost-effectiveness. Moreover, this model contributes to our fundamental understanding of tumor development by evaluating the activity of individual mechanisms and the expression of individual proteins in different grades, thus allowing us to derive useful information regarding the mechanisms involved in carcinogenesis and potential therapeutic targets.

## Materials and methods

### Data collection and processing

RNA-seq and microarray data for BRCA, LUAD, and ccRCC were collected from multiple datasets (**Supplementary Table 1**). RNA-seq data from TCGA and PCAWG datasets were downloaded from the Xena data hub (72). Data on somatic mutations and proteomes for these datasets were downloaded from the Xena data hub. ABSOLUTE (RRID: SCR_005198) segments for TCGA [as in supplemental data for TCGA - PanCanAtlas Publications (73)] were downloaded from the GDC. The PCAWG segment data were downloaded from Xena. Survival data were downloaded from the Xena data hub. Original grade labels for TCGA-BRCA were obtained from pathological reports on the GDC data portal. CPTAC-3 RNA-seq data and survival annotations were downloaded from the GDC data portal. Additionally, a list of CPTAC kidney carcinomas with clear cell histology was curated from the National Institutes of Health Proteomic Data Commons. RNA-seq data for GSE96058 and GSE68417 were downloaded from the GEO database. All other GEO array datasets were downloaded from GEO as raw data (CEL files or other formats) and processed using gcrma or oligo followed by limma with default settings. Survival and grade annotations were curated from GEO annotations.

### Rank transformation

Rank transformation was employed to mitigate batch effects while preserving the relationships between genes. For each sample, genes were ranked by their expression level and ties were assigned the average ranks. Missing values were omitted from the analysis. For example, for a geneset of N (where N represents the number of genes in the geneset for a particular sample):


$$Expression_{Geneset\;A}=\left[x_{1},x_{2},x_{3}\ldots x_{N-1},\;x_{N}\;\right]\;\Rightarrow^{in\;ascending\;order\;}\Rightarrow$$

$$\Rightarrow \left[x_{2,\;}x_{15},x_{N-1}\ldots x_{1},\;x_{N}]\;\Rightarrow \;[1,\;2,\;3\ldots \;N-1,\;N\right]$$

This transformation ensured consistent results across different datasets without the need for batch-specific corrections, due to its ability to preserve relationships like “Gene A > Gene B” while transforming data into fixed and stable range of values independent of cohort composition. The ranked gene expression values were subsequently used for further machine-learning analysis. Decision trees (and, consequently, gradient-boosted trees) that rely on comparisons of Gene A to some value X benefit from this approach because value X is now reflective of the position of Gene A in the geneset analyzed and is generally preserved by this transformation method even when derived from different data batches.

### Machine learning

Gradient boosted trees (LightGBM, version 4.5.0) were trained as follows. A five-fold cross-validation procedure was applied using stratified sampling to maintain the balance of the mGrade labels across the training and test sets (80% training and 20% testing). At each step of the iterative refinement, the best classifier hyperparameters were selected based on the best negative log-loss score, and the quality was assessed for each split. Subsequently, a classifier was trained with these selected hyperparameters, and the SHAP values were calculated. The two least important genes according to the SHAP values were discarded, and the procedure was repeated until 20 genes remained. The final geneset was then chosen based on the best mean negative log-loss score and its deviation recorded during the five-fold cross-validation for each step (See **Figure 1**). The final classifier hyperparameters were optimized based on the negative log-loss score during cross-validation. The following parameters were inherently different for each model. Parameters for the BRCA model were: n_esimators=500, reg_alpha=0.3, reg_lambda=0.6, max_depth=-1. Parameters for the LUAD model were: n_esimators=500, reg_alpha=0, reg_lambda=0.6, max_depth=-1. Parameters for the ccRCC model were: n_esimators=200, reg_alpha=0, reg_lambda=0.6, max_depth=-1. All other parameters remained unchanged and in default settings.

### Differential gene expression analysis

Differential gene expression analysis was performed using limma for the microarray data and edgeR for the RNA-seq data. Genes with q-value < 0.05, |logFC| > 1 (logFC > 1.5 or logFC < -1.2 for LUAD) and a minimum logCPM of 2 (RNA-seq), or average expression > 3 (breast microarray), or average expression > 7 (kidney microarray) were selected for further analysis. The analysis compared high-grade tumors (G3/G4) with low-grade tumors (G1) across all cancer types.

### Grade labeling

Validation of mGrades was conducted by comparing the predicted grades with the histological grades using datasets from TCGA, CPTAC, and GEO. For ccRCC, the Fuhrman grading system was used for validation. For BRCA and LUAD, the Nottingham Histological Grade and WHO classification criteria were used, respectively. BRCA grade labels were curated from pathological reports, whereas grade labels for LUAD and ccRCC were assessed from histological images according to established guidelines. For consistency in machine-learning labels, we defined low-grade = G1 and high-grade = G3 ± G4, treating any available G4 cases as part of the high-grade pool. In BRCA and LUAD public datasets, true G4 specimens are rare and biologically overlap with G3; lumping them with G3 therefore maximizes sample size without diluting biological signal. In ccRCC, G4 constitutes a recognized aggressive category and was likewise merged with G3 to form a single “high-grade” class. Following the differential gene expression analysis described above, a GGI-like index was constructed for each sample. This unscaled index was used in a subsequent Cox regression survival analysis to determine the optimal threshold that best separated patients into high- and low-risk groups [similar to the original GGI approach (21)].

### Pathway analysis

To identify enriched pathways, we conducted a hypergeometric test on selected genes, focusing on the Canonical Pathways MSigDB Collections (Hallmark 50 (74), KEGG (RRID: SCR_012773) (75), WikiPathways (RRID: SCR_002134) (76), and REACTOME (RRID: SCR_003485) (77) as examples of the most prominent collections in the list). Additionally, plots of the enriched REACTOME pathways in mG3 were generated using ReactomePA. Pathway activity scores were calculated using ssGSEA (GSVA package) (78).

### Survival analysis

Cox regression analysis was performed to assess the association between mGrades and patient survival using Python lifelines package v.0.30 (RRID: SCR_024202) (79). The stage (lymph node status for BRCA) and cohort characteristics were accounted for, with significance determined at p < 0.05. Proportional hazard assumption was tested using Python lifelines package v.0.30 for all survival variables in the validation sets. The only survival variable presenting p-values < 0.05 was GSE25066 in BRCA (p = 0.0058). However, when only a subset of G2 samples within the dataset were assessed to determine their split into either mG1 or mG3, the p-value became > 0.05. This deviation within GSE25066 likely resulted from the difference in the number of samples tested and thus was deemed insignificant. Integrated Brier scores were calculated with scikit-survival version 0.24.1 between 100 days and 1,700 days post diagnosis.

### Analysis of features correlated with mGrades

Chi-square tests were used to evaluate the correlation between mGrades and clinical stages. For the analysis of differential mutations, the following filtering criterion was first applied: Tumor Variant Allele Frequency > 0.05. Genetic variants that met this criterion were selected. The following variant classifications were then excluded: Intron, 3’Flank, 5’Flank, and Synonymous_Mutation. Before conducting the tests, cutoffs depending on the total number of samples were set to retain only highly mutated genes (10–15 genes for BRCA and LUAD; five genes for ccRCC due to a lower number of highly mutated genes in the test cohort). *TTN*, *MUC16*, and *RYR2* were excluded from testing because of their high somatic mutation frequencies associated with larger lengths. Differential mutation analysis between mGrades was conducted using Fisher’s exact test. FDR correction was applied and the q-value threshold was set at < 0.05. LogFC for the drawing of volcano plots and effect size representation was defined as the log of the percentage of mutated samples in mG3 divided by the percentage of mutated samples in mG1.

CNA segments were normalized by subtracting sample ploidy from the total copy number and clipped to a range of -2 to 2. Segments were then intersected with cytoband annotations using bedtools (RRID: SCR_006646). The resulting cytobands were considered as deleted or amplified when the normalized copy number was -2 or 2, respectively. The deletions and amplifications were tested separately. Cytobands with a low number of alterations were discarded before testing. Differential cytoband analysis between the mGrades was conducted using Fisher’s exact test. FDR correction was applied and the q-value threshold was set at < 0.05. LogFC was calculated as described for the mutation analysis.

Differential protein analysis was conducted using the Mann–Whitney *U* test to compare protein expression between groups. The p-values were FDR-corrected.

The Mann–Whitney *U* test was used to assess differences in pathway activities between mGrades. The p-values were also FDR-corrected.

## Data Availability

The original contributions presented in the study are included in the article/**Supplementary Material**. Further inquiries can be directed to the corresponding author.

## Supplementary material

Supplementary Figure 1 Relabeling of train datasets with mGrades using the GGI index. 1st row: GGI-like index vs. pathologist-labeled grades. 2nd row: GGI distribution in the training datasets with the chosen cutoff between high and low mGrades (in red). 3rd row: Cox regression analysis of the training datasets with the chosen cutoff between mGrades. 4th row: Cox regression analysis of the training datasets with pathologist-labeled grades.

Supplementary Figure 2 Refinement of the classifier feature set. **(A)** Feature selection process. Mean and standard deviation of negative log loss for each number of features remaining in training. **(B)** The 20 most important features of the final classifier and their SHAP values for each sample in the training dataset.

Supplementary Figure 3 Mutations and CNA analysis in mGrades. **(A)** Volcano plots depicting differential mutations. **(B)** Volcano plots depicting differential CNA cytobands. ccRCC samples are not shown because their results are not significant. Right, cytoband deletions; left, cytoband amplifications.

Supplementary Figure 4 Pathway activity and enrichment in mGrades. **(A)** Volcano plots depicting differential pathway activities. **(B)** Reactome enrichment of genes upregulated in mG3 based on classifier-chosen genesets.
