# Supplementary figures and images for: Machine learning-based single-sample molecular classifier for cancer grading
Source: Front Oncol. 2025 Jul 16;15:1617898. doi: 10.3389/fonc.2025.1617898 (PMC12307393; doi:10.3389/fonc.2025.1617898)

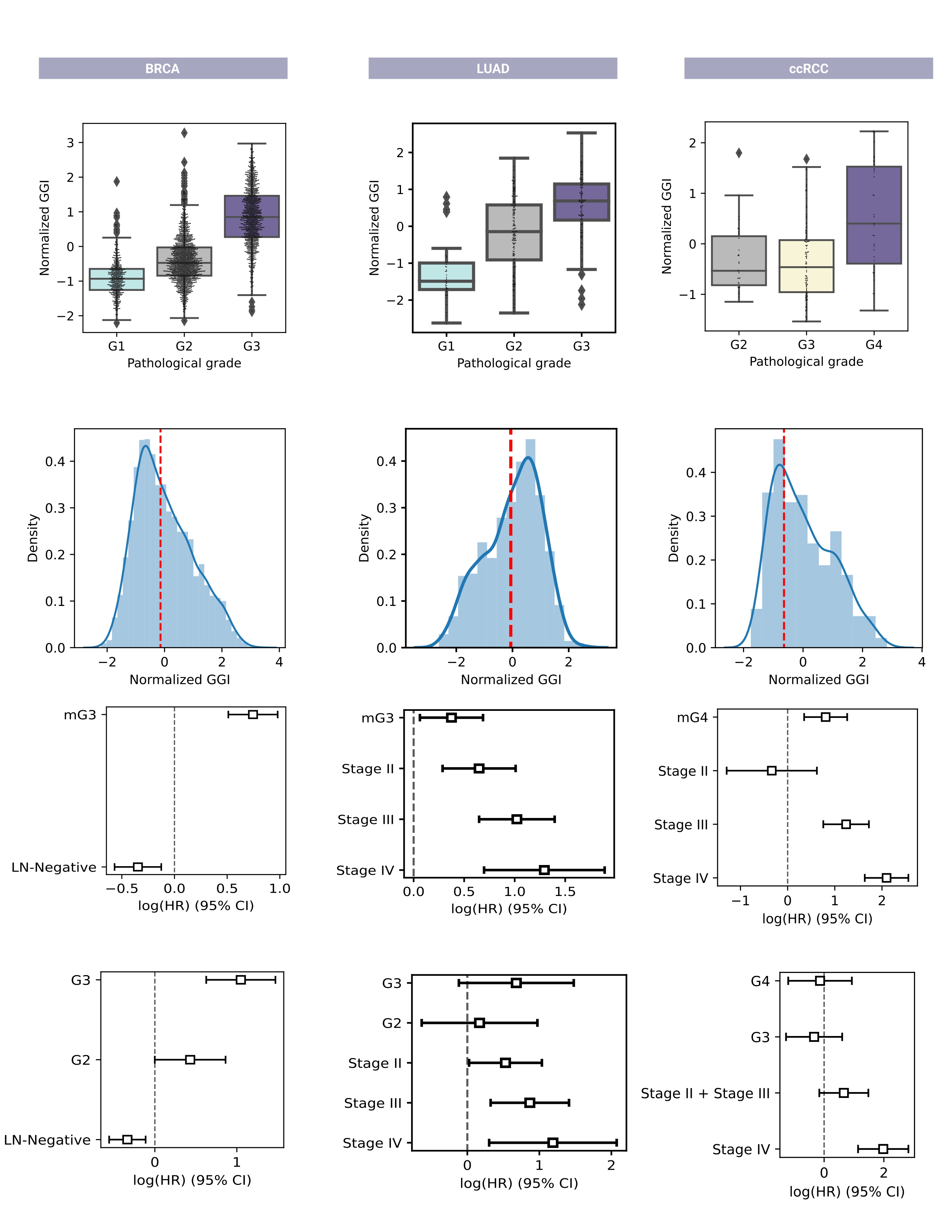

Supplement: Supplementary Figure 1 — Relabeling of train datasets with mGrades using the GGI index. 1st row: GGI-like index vs. pathologist-labeled grades. 2nd row: GGI distribution in the training datasets with the chosen cutoff between high and low mGrades (in red). 3rd row: Cox regression analysis of the training datasets with the chosen cutoff between mGrades. 4th row: Cox regression analysis of the training datasets with pathologist-labeled grades. [file Image1.jpeg]

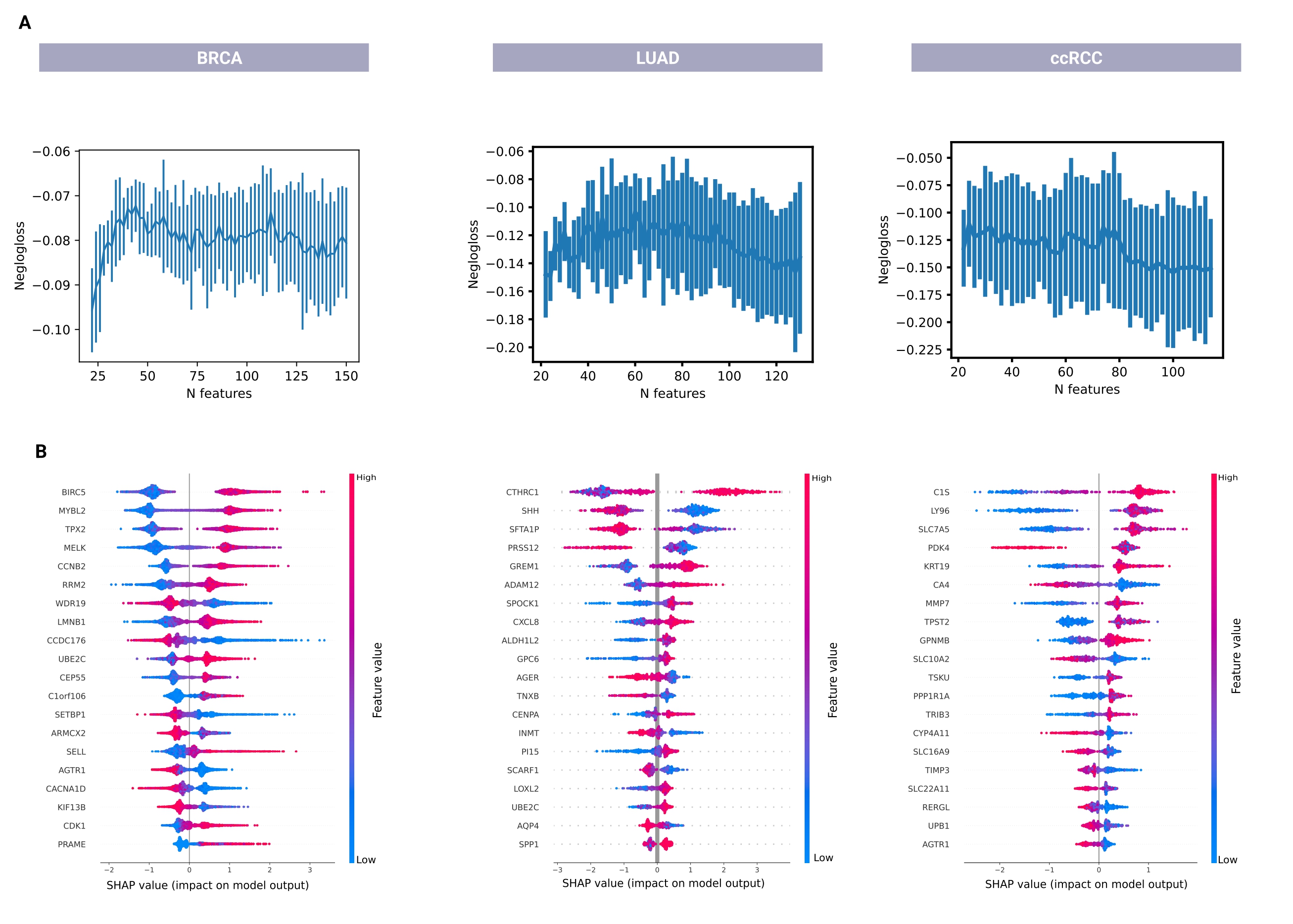

Supplement: Supplementary Figure 2 — Refinement of the classifier feature set. (A) Feature selection process. Mean and standard deviation of negative log loss for each number of features remaining in training. (B) The 20 most important features of the final classifier and their SHAP values for each sample in the training dataset. [file Image2.jpeg]

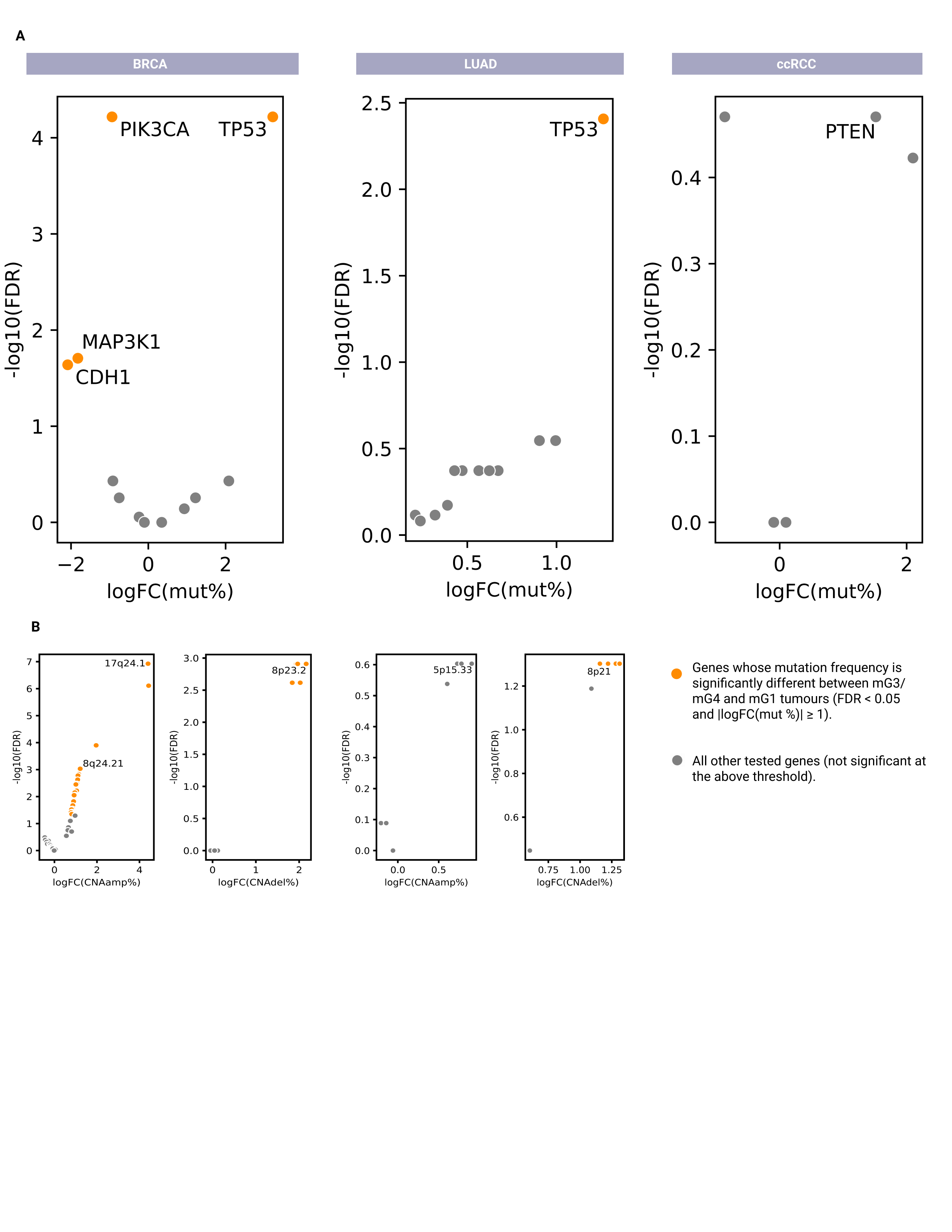

Supplement: Supplementary Figure 3 — Mutations and CNA analysis in mGrades. (A) Volcano plots depicting differential mutations. (B) Volcano plots depicting differential CNA cytobands. ccRCC samples are not shown because their results are not significant. Right, cytoband deletions; left, cytoband amplifications. [file Image3.jpeg]

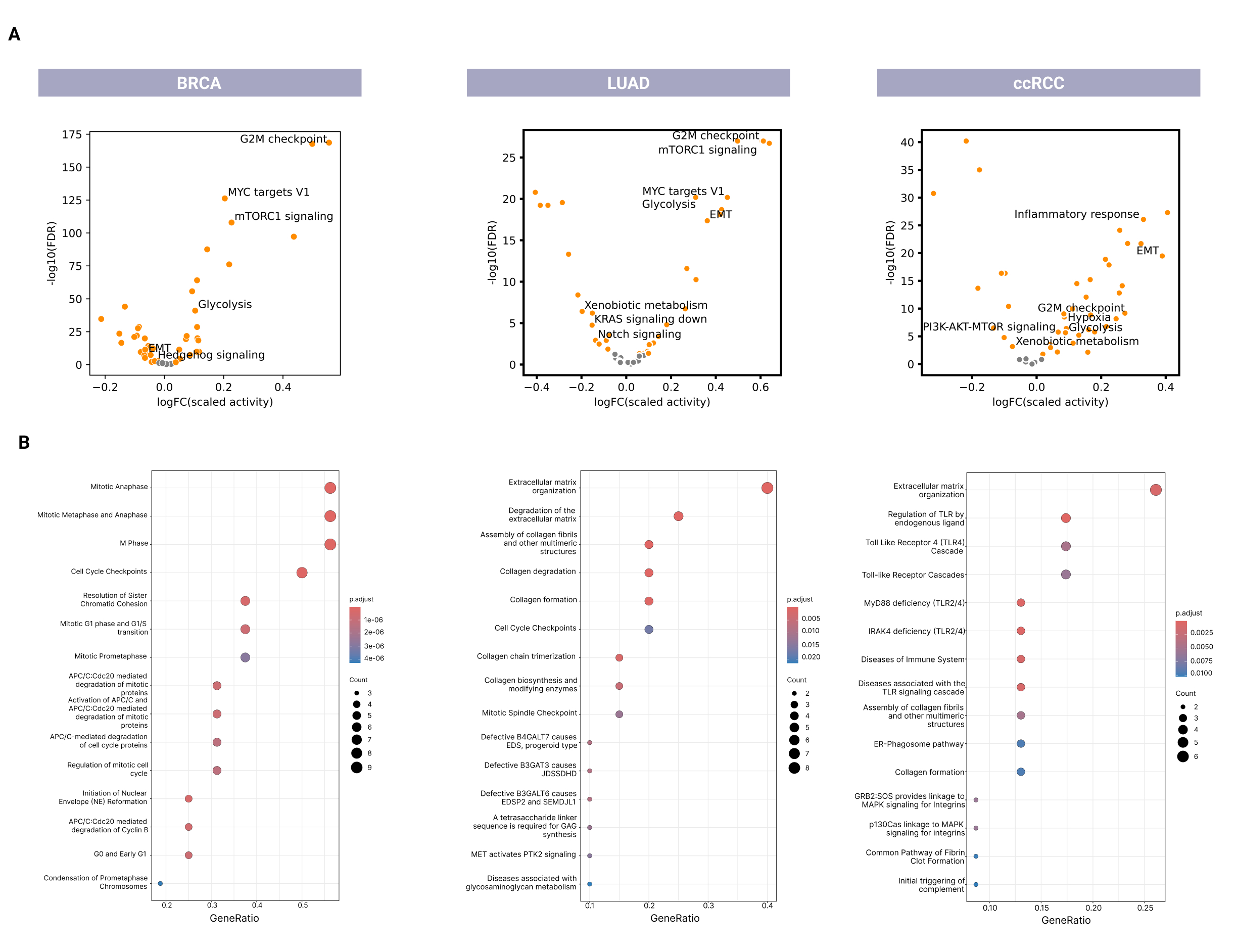

Supplement: Supplementary Figure 4 — Pathway activity and enrichment in mGrades. (A) Volcano plots depicting differential pathway activities. (B) Reactome enrichment of genes upregulated in mG3 based on classifier-chosen genesets. [file Image4.jpeg]
